# Changes in Density of On-Premises Alcohol Outlets and Impact on Violent Crime, Atlanta, Georgia, 1997–2007

**DOI:** 10.5888/pcd12.140317

**Published:** 2015-05-28

**Authors:** Xingyou Zhang, Bonnie Hatcher, Lydia Clarkson, James Holt, Suparna Bagchi, Dafna Kanny, Robert D. Brewer

**Affiliations:** Author Affiliations: Bonnie Hatcher, Lydia Clarkson, Suparna Bagchi, Chronic Diseases, Healthy Behaviors and Injury Epidemiology Section, Georgia Department of Public Health, Atlanta, Georgia; James Holt, Dafna Kanny, Robert D. Brewer, National Center for Chronic Disease Prevention and Health Promotion, Centers for Disease Control and Prevention, Atlanta, Georgia.

## Abstract

**Introduction:**

Regulating alcohol outlet density is an evidence-based strategy for reducing excessive drinking. However, the effect of this strategy on violent crime has not been well characterized. A reduction in alcohol outlet density in the Buckhead neighborhood of Atlanta from 2003 through 2007 provided an opportunity to evaluate this effect.

**Methods:**

We conducted a community-based longitudinal study to evaluate the impact of changes in alcohol outlet density on violent crime in Buckhead compared with 2 other cluster areas in Atlanta (Midtown and Downtown) with high densities of alcohol outlets, from 1997 through 2002 (preintervention) to 2003 through 2007 (postintervention). The relationship between exposures to on-premises retail alcohol outlets and violent crime were assessed by using annual spatially defined indices at the census block level. Multilevel regression models were used to evaluate the relationship between changes in exposure to on-premises alcohol outlets and violent crime while controlling for potential census block-level confounders.

**Results:**

A 3% relative reduction in alcohol outlet density in Buckhead from 1997–2002 to 2003–2007 was associated with a 2-fold greater reduction in exposure to violent crime than occurred in Midtown or Downtown, where exposure to on-premises retail alcohol outlets increased. The magnitude of the association between exposure to alcohol outlets and violent crime was 2 to 5 times greater in Buckhead than in either Midtown or Downtown during the postintervention period.

**Conclusions:**

A modest reduction in alcohol outlet density can substantially reduce exposure to violent crime in neighborhoods with high density of alcohol outlets. Routine monitoring of community exposure to alcohol outlets could also inform the regulation of alcohol outlet density, consistent with *Guide to Community Preventive Services* recommendations.

## Introduction

Excessive alcohol use is responsible for approximately 88,000 deaths and 2.5 million years of potential life lost in the United States each year (1); excessive alcohol consumption accounted for $223.5 billion in economic costs ($1.90/drink) in 2006 (2). Excessive alcohol use is also associated with many health and social problems, including violence (3).

A large body of literature describes the relationship between alcohol outlet density and adverse population health outcomes, including violent crime (4–7). The Community Preventive Services Task Force (CPSTF) recommended regulating alcohol outlet density on the basis of “evidence of a positive association between outlet density and excessive alcohol consumption and related harms” (8). However, the trend in the United States is to deregulate alcohol sales (eg, privatize retail alcohol sales), often resulting in substantial increases in alcohol outlet density (9). Therefore, most studies have focused on the impact of increasing alcohol outlet density on various health outcomes, including violent crime. With few exceptions (10–13), studies assessing the relationship between alcohol outlet density and violence, particularly violent crime, are cross-sectional and therefore cannot evaluate the potential impact of changes in alcohol outlet density on violent crime. Furthermore, many studies assessing the relationship between changes in alcohol outlet density and violent crime had important methodological limitations. First, many of these studies were based on changes in the numbers of alcohol outlets and violent crimes within a geopolitical unit (ie, city, county, or zip code). The limitation with this “container-based approach” is that retail alcohol outlets and violent crime are not evenly distributed or restricted to a geopolitical area. Retail alcohol outlets and violent crime commonly are clustered in specific areas in a community (14) and may cross area boundaries (15). Consequently, an assessment of outlet density and crime that is restricted by boundaries may miss important geospatial relationships, because some of these exposure–outcome relationships are not assessed or the relationships are diluted over a larger area. To overcome this limitation, it is important to assess the relationship between alcohol outlet density and crime on the basis of spatial clustering and not simply the colocation of outlets and crime in the same general area.

The second limitation is that many studies compare areas with high alcohol outlet density to areas with low alcohol outlet density. Because many factors, in addition to alcohol outlet density, can affect violent crime (16,17), this approach makes it difficult to evaluate the effect of changes in outlet density on violent crime independent of other environmental factors (eg, differences in law enforcement and economic development). To overcome this limitation, it is important to compare areas with high alcohol outlet density to one another and then observe how changes in this environmental exposure in one area affects violent crime relative to other areas with similar outlet density that may not have experienced similar changes in the retail environment.

A reduction in alcohol outlet density in Buckhead, an affluent mixed (residential and commercial) neighborhood in northeast Atlanta with a high clustering of on-premises alcohol outlets (ie, bars and clubs), provided an opportunity to assess the relationship between an observed substantial reduction in alcohol outlet density and violent crime. After a period of significant growth in the number of on-premises retail alcohol outlets and several high-profile homicides and assaults in Buckhead during the 1990s, community residents requested that the Atlanta mayor and city council establish and enforce restrictions on retail sales of alcohol in Buckhead. Many of these restrictions were consistent with CPSTF recommendations for preventing excessive alcohol consumption and related harms (8) and included restricting the hours when alcohol could be sold (18) and enforcing laws prohibiting alcohol sales to minors (19). These changes in the retail alcohol sales environment occurred before or were coincident with the closure of many on-premises alcohol outlets in Buckhead and occurred before the sale of these properties for development as residential and retail space (20–27).

The purpose of this study was to assess whether the observed reduction in the density of alcohol outlets in Buckhead was associated with a reduced exposure to violent crime during the same period.

## Methods

A community-based longitudinal study was conducted to evaluate the impact of changes in alcohol outlet density on violent crime in the intervention area, Buckhead, compared with the 2 control areas, Midtown and Downtown, which also had high clusters of alcohol outlets (ie, a large number of on-premises alcohol outlets located in a small geographic area), from 1997 through 2007. This block-level analysis allowed for a precise assessment of the environmental impact of changes in alcohol outlet density on exposure to violent crime and thus the safety of various neighborhoods independent of whether the people living in the neighborhoods had themselves experienced violent crime. Many changes in the alcohol retail sales environment in the Buckhead area occurred during or before 2003; therefore, the preintervention period was defined as from 1997 through 2002, and the postintervention period as from 2003 through 2007. This period was selected to capture the increase in alcohol outlet density that occurred before 2003, the decrease that occurred between 2003 and 2007, and the effect these changes had on violent crime. This study was exempt from internal review board approval because personal identifiers were not included in the data provided to the Georgia Department of Public Health and the Centers for Disease Control and Prevention (CDC).

### Data sources

Alcohol licensure data for retail alcohol establishments in Atlanta from 1997 through 2007 were obtained from the Georgia Department of Revenue. These data included the type of establishment (ie, on-premises vs off-premises) and street address. Data for Federal Bureau of Investigation (FBI) class I violent crimes — including homicide, rape, robbery, and aggravated assault — that occurred from 1997 through 2007 were obtained from the Atlanta Police Department. These data included type, date, time, and location (street address) of the crime. On-premises retail alcohol outlets and violent crimes were geocoded by the Georgia Department of Public Health using Centrus Desktop version 4.1 (Pitney Bowes Co).

Demographic information on age (0–14 y, 15–34 y, ≥ 35 y), sex, race/ethnicity (% white, black, Hispanic, other), and poverty rate (% living below the federal poverty level) of Atlanta residents were obtained at the census block group level from the US 2000 Census. High, medium, and low poverty census blocks corresponded to areas where more than 34.4% (fourth quartile), more than 8.1% to no more than 34.4% (second and third quartiles), and 8.1% or less (first quartile) of the residents lived in households with incomes below the federal poverty level.

**Figure 1:**
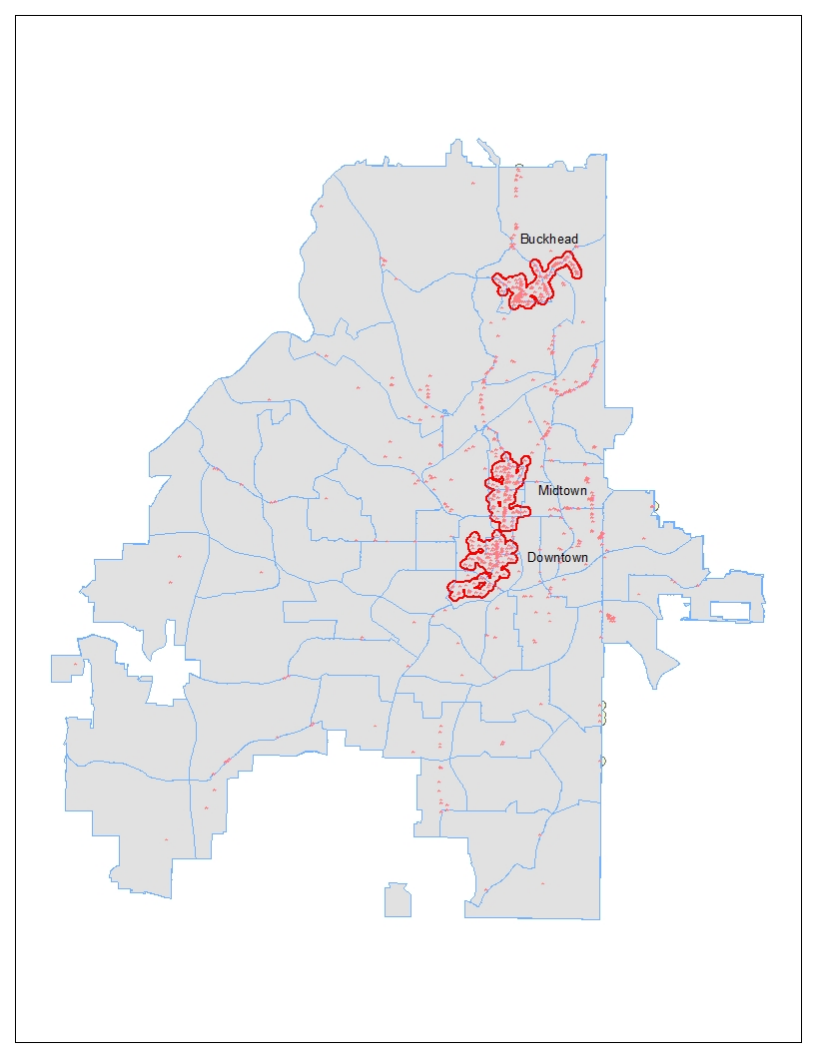
On-premises alcohol outlets and cluster zones in Atlanta, GA, 1997–2007.

Based on an exploratory spatial analysis, more than 90% of the on-premises retail alcohol outlets in Atlanta were located within 0.1 mile of another outlet; therefore, this distance was used to create a buffer around each outlet throughout the city. Overlapping buffers were merged into larger alcohol outlet clusters if the distance between them was 0.1 mile or less. High-density clusters of alcohol outlets were defined as areas that contained 50 or more on-premises retail alcohol outlets located within 0.1 mile or less of at least 1 other retail alcohol establishment. Buckhead (0.90 square miles), Midtown (0.95 square miles), and Downtown (1.09 square miles) Atlanta were identified as having high-density clusters of alcohol outlets (Figure 1). All census blocks in the 3 high-density clusters were included in this study: 35 in Buckhead, 109 in Midtown, and 210 in Downtown. Because these 3 areas were defined on the basis of density and spatial considerations only, they did not necessarily correspond to established administrative boundaries that might be used elsewhere.

### Data analysis

Exposure to on-premises retail alcohol outlets for each census block was assessed by using an annual alcohol outlet exposure index. This approach was used to evaluate the physical availability of alcohol and the potential for second-hand effects that could be related to alcohol availability (eg, interpersonal violence) at the census block level. We used the sum of the inverse distances between a neighborhood census block centroid and the nearest 7 alcohol outlets during a specific year to calculate the index. The decision to focus on the nearest 7 outlets as opposed to all outlets in an area was based on cognitive studies that have shown that 7, plus or minus 2, is generally the maximum number of options that individuals (in this case, potential patrons, not residents) are likely to consider when making choices or evaluating environmental conditions (28). To obtain an annual alcohol outlet exposure index for each cluster, we summed annual alcohol outlet exposure indices across all the census blocks in each high-density cluster of alcohol outlets weighted by the population residing in each census block to account for heterogeneous population distributions within each cluster.

We used an annual violent crime exposure index to assess exposure to violent crime for each census block. As with the analysis of alcohol outlet exposure, the annual violent crime exposure index was calculated by using the sum of the inverse distances between a census block centroid and the nearest 7 violent crime events during a specific year. To obtain an annual violent crime exposure index for each cluster, we summed violent crime exposure indices across all the census blocks in each high-density cluster of alcohol outlets weighted by the population residing in each census block to account for heterogeneous population distributions within each cluster.

Multilevel regression was used to evaluate the relationship between changes in census block-level alcohol outlet exposure indices and violent crime exposure indices in the Buckhead (intervention) cluster relative to the Midtown and Downtown (control) clusters, while controlling for potential confounders (age, sex, race/ethnicity, and poverty of neighborhood residents at the census block group level). The model included a random intercept, whose mean represented the overall average of violent crime exposure indices for all census blocks under study during 2002, and a random slope, whose mean represented the overall rate of change in the violent crime exposure indices for all census blocks under study during the entire study period (1997–2007). Significance was assessed at the *P* < .05 level. Block-level spatial autocorrelations were also explored.

## Results

We obtained 7,879 on-premises alcohol license records from the City of Atlanta for 1997 through 2007. Of these, 7,450 (94.6%) were geocoded and included in the study. During this same period, there were 263,379 class I FBI crimes in Atlanta. Of these, 194,247 crimes (73.8%) were geocoded, and 188,138 (71.4%) occurred in the City of Atlanta. Of these 188,138 violent crimes, 17,215 (9.2%) occurred in 1 of the 3 clusters: 2,206 in Buckhead; 4,456 in Midtown; and 10,553 in Downtown. The distribution of violent crimes by type of offense varied across the 3 clusters, but most of these crimes (>98%) were either aggravated assaults or robberies (Table 1).

**Table 1:** Selected Characteristics of Cluster Zones of On-Premises Alcohol Outlets, Atlanta, Georgia, 1997–2007

Characteristics^a^	Atlanta	Cluster Zones		
		Buckhead	Midtown	Downtown
Census 2000 population, N	416,474	1,498	7,878	4,653
Area, sq mi	132.4	0.90	0.95	1.09
**Age group, y**				
0–14	19.0	6.3	2.3	6.0
15–34	36.3	46.7	57.2	49.3
≥35	44.7	46.9	40.5	44.7
**Sex**				
Male	49.6	49.6	66.0	68.5
Female	50.4	50.4	34.0	31.5
**Race/ethnicity**				
White	31.3	74.6	63.7	26.2
Black	61.0	7.4	24.7	65.1
Hispanic	4.5	9.4	4.2	5.1
Other	3.2	8.6	7.5	3.6
**Poverty^b^**				
High	26.0	0.0	21.9	77.6
Medium	51.2	82.8	70.0	22.4
Low	22.8	17.2	8.2	0.0
**On-premises alcohol outlets (N)^c^**	7,450	1,107	1,142	1,378
**Violent crime records (N)^c^**	188,138	2,206	4,456	10,553
Aggravated Assaults	74.9	76.5	64.1	68.4
Robberies	22.6	21.6	33.9	29.8
Homicide	0.8	0.4	0.4	0.4
Other	1.7	1.5	1.6	1.4

a Values are percentages unless otherwise indicated.

b High poverty defined as census block group level poverty greater than 34.4% (fourth quartile); medium poverty defined as census block group level poverty rate greater than 8.1% but no more than 34.4% (second and third quartiles); low poverty defined as census block group level poverty 8.1% or less (first quartile).Percentage of population residing in high, medium, or low poverty census block groups.

c On-premises alcohol outlets and violent crime records are the total counts for 1997–2007

The number of on-premises alcohol outlets in Buckhead increased from 98 in 1997 to 111 in 2001, and then declined to 87 in 2007. In contrast, the number of on-premises alcohol outlets in Midtown increased from 85 in 1997 to 122 in 2007, and the number of on-premises alcohol outlets in Downtown increased from 109 in 1997 to 152 in 2007.

**Figure 2:**
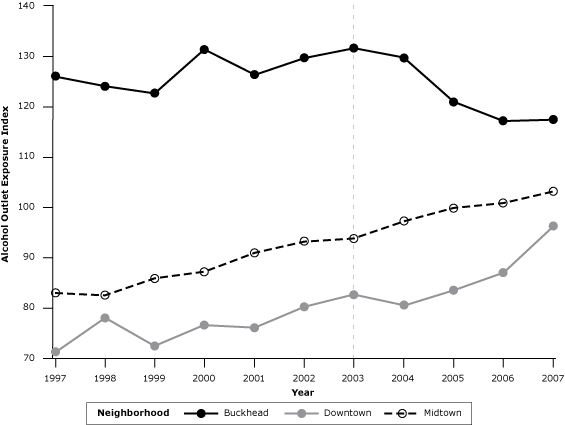
On-premises alcohol outlet exposure indices by neighborhood, Atlanta, Georgia, 1997–2007. This graph shows the temporal change in spatial exposure to on-premises alcohol outlets from 1997–2007 for 3 Atlanta neighborhoods: Buckhead, Downtown, and Midtown. **Table:** 

Consistent with the changes in the number of on-premises outlets, the annual alcohol outlet exposure index in Buckhead initially increased during the preintervention period from 1997 through 2002, remained constant during the early part of the intervention period, and began to decrease from 2003 through 2007, resulting in an overall decline of 6.8% during the study period (Figure 2), with the greatest decline occurring after 2003. In contrast, the alcohol outlet exposure index consistently increased throughout the study period in both control clusters, resulting in an overall increase of 24.4% in Midtown and 35.2% in Downtown. The relative change in the alcohol outlet exposure index from the preintervention (1997–2002) to postintervention (2003–2007) periods was −3.2% in Buckhead, 12.1% in Midtown, and 12.4% in Downtown.

Year	Alcohol Outlet Exposure Index		
	Buckhead	Midtown	Downtown
1997	126.0	83	71.3
1998	124.1	82.6	78.0
1999	122.7	85.9	72.4
2000	131.4	87.2	76.6
2001	126.3	91.0	76.1
2002	129.6	93.2	80.3
2003	131.6	93.8	82.7
2004	129.6	97.3	80.6
2005	120.9	99.9	83.6
2006	117.2	100.9	87.1
2007	117.4	103.2	96.4

**Figure 3:**
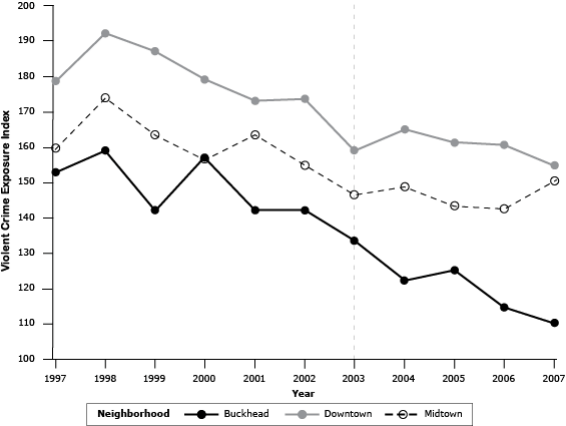
Violent crime exposure indices by neighborhood, Atlanta, Georgia, 1997–2007, showing the temporal change in violent crime exposure from 1997–2007 for 3 Atlanta neighborhoods: Buckhead, Downtown, and Midtown. **Table:** 

During this same period, the violent crime exposure indices decreased in all 3 clusters; however, the decrease was substantially greater in Buckhead (28%) than in either Midtown (6%) or Downtown (13%) (Figure 3). Comparing the preintervention and postintervention periods, the relative decline in the violent crime exposure index was about twice as great in Buckhead (−17.5%) than in either Midtown (−8.4%) or Downtown (−9.8%).

Year	Violent Crime Exposure Index		
	Buckhead	Midtown	Downtown
1997	152.8	159.8	178.8
1998	159.2	174	192.3
1999	142.3	163.5	187.3
2000	157.1	156.6	179.2
2001	142.2	163.6	173.2
2002	142.2	154.8	173.7
2003	133.6	146.7	159.1
2004	122.4	148.9	165.2
2005	125.4	143.4	161.3
2006	114.7	142.7	160.8
2007	110.2	150.4	154.9

The model comparison showed that a multilevel spatial model had a higher Akaike information criterion (AIC) (45,716) than the one in this study (AIC = 45,686). Thus, block-level spatial autocorrelation was not a significant problem for this data set. Stronger block-level intraclass correlation was observed for the violent crime exposure index (intraclass correlation coefficient) = 0.65).

After adjusting for confounding factors, the alcohol outlet exposure index remained significantly associated with the violent crime exposure index in all 3 clusters (Table 2). However, during the preintervention period (1997–2002), the impact of exposure to alcohol outlets on exposure to violent crime was 2 to 4 times greater in Buckhead (regression coefficient [RC] = 0.84) than in either Midtown (RC = 0.18) or Downtown (RC = 0.32). Similarly, during the postintervention period (2003–2007), the impact of exposure to alcohol outlets on exposure to violent crime was about 2 to 5 times greater in Buckhead (RC = 0.65) than in either Midtown (RC = 0.12) or Downtown (RC = 0.27).

**Table 2:** Multilevel Regression Coefficients for Violent Crime Exposure by Cluster Zones of On-Premises Alcohol Outlets, Atlanta, Georgia, 1997–2007

Fixed Effects	Coefficient Estimate (Standard Error)	*P* Value
**Intercept**	170.99 (29.04)	<.001
**Year (centered at 2002)**	−2.55 (0.64)	<.001
**Black, %**	0.23 (0.45)	.62
**Male (15–34 y), %**	−0.50 (0.36)	.17
**Poverty**		
High	−4.60 (13.21)	.73
Medium	Reference	
Low	−58.54 (24.76)	.02
**Cluster zone**		
Buckhead	−67.46 (26.64)	.011
Midtown	Reference	
Downtown	12.08 (20.80)	.56
**Alcohol outlet exposure**		
Buckhead preintervention	0.84 (0.13)	<.001
Buckhead postintervention	0.65 (0.12)	<.001
Midtown preintervention	0.18 (0.05)	.001
Midtown postintervention	0.12 (0.05)	.02
Downtown preintervention	0.32 (0.08)	.001
Downtown postintervention	0.27 (0.08)	.004

## Discussion

To our knowledge, this is the first study to use geospatially defined exposure indices to characterize both alcohol outlet density and exposure to violent crime at the census block level, and to assess changes in this association over time. The results of this study indicate that a modest (3.2%) relative reduction in on-premises alcohol outlet density in the Buckhead area from 1997–2002 to 2003–2007 was associated with a 2-fold greater reduction in exposure to violent crime in the neighborhoods surrounding these alcohol outlets than in the neighborhoods surrounding other high-density clusters of alcohol outlets (Midtown and Downtown) where exposure to on-premises retail alcohol outlets increased. The relationship between exposure to alcohol outlets and violent crime was 2 to 5 times greater in Buckhead than in the control areas during the postintervention period, even after controlling for other factors that could influence violent crime rates (ie, age, sex, race/ethnicity, and household income of area residents), suggesting that the reduction in alcohol outlet density in Buckhead was a major contributor to the significantly greater reduction in exposure to violent crime that occurred in Buckhead relative to the control neighborhoods.

The findings of this study are consistent with those of longitudinal studies that assessed the impact of reducing alcohol outlet density on violent crime (10–12). One study in Los Angeles, California, found that the closure of retail alcohol outlets following civil unrest in early 1992 resulted in lower assault rates in affected areas than in areas in the city that did not experience a reduction in alcohol outlets (12). These findings emphasize the strong spatiotemporal relationship between retail alcohol outlets and violent crime in local neighborhoods (14,15).

The stronger association between exposure to retail alcohol outlets and violent crime in the Buckhead area than in the Midtown and Downtown areas is probably due to the higher alcohol outlet exposure index in Buckhead than in either of these 2 control clusters throughout the study period. High alcohol outlet-density is an environmental risk factor for excessive alcohol consumption, which is a risk factor for interpersonal violence (29,30). In addition, high alcohol outlet density can aggregate excessive drinkers in a small geographic area, further increasing the risk of interpersonal violence. High alcohol outlet density can also increase competition between alcohol retailers, leading to more aggressive alcohol marketing, including volume discounts and point-of-purchase alcohol advertising, which can further increase the risk of excessive alcohol use (30).

Our study focused on neighborhoods that were directly exposed to high concentrations of retail alcohol outlets, which had the greatest potential for exposure to harm that could be related to it (eg, interpersonal violence). This approach also improved the sensitivity and precision of our analysis by allowing us to control for factors that may differentiate areas with high alcohol outlet density from other areas in the city (ie, to select areas with increased alcohol consumption, increased visitors and traffic, and increased law enforcement). This study design also avoided the classic “container-based approach” that can occur when analyzing alcohol outlet density and violent crime by using convenient or prescribed geographic units (eg, census tracts, zip codes, police zones, neighborhood planning units), which may not take into account the uneven distribution of these exposures in neighborhoods, and consequently hide areas with high alcohol outlet density and violent crime that cross geopolitical boundaries.

This study had several limitations. First, the alcohol licensure data in this study could not differentiate nightclubs or bars from other on-premises retail alcohol outlets (eg, restaurants). Second, violent crime was likely to have been underreported. An audit of the Atlanta Police Department’s 2002 crime data found that Uniform Crime Report Part I Crimes (ie, the violent crimes that were assessed in this study) were underreported by 3.2%, and some of these crimes were misclassified as lesser offenses (25). Finally, this study is ecological, and it is not possible to definitively state that the greater reduction in violent crime in Buckhead relative to the 2 control neighborhoods was due to changes in alcohol outlet density alone. However, the strong temporal relationship between the reduced exposure to alcohol outlets and violent crime at the census block level in Buckhead relative to the control neighborhoods provides strong presumptive evidence that the reduction in violent crime was due, at least partly, to the reduction in alcohol outlet density in this area. This conclusion is further supported by the known relationship between alcohol outlet density and violent crime that was assessed by CPSTF (30).

A modest 3% reduction in on-premises alcohol outlet density (assessed using an alcohol outlet exposure index) can substantially reduce exposure to violent crime in neighborhoods that have high alcohol-outlet density, particularly when compared with other urban areas where alcohol outlet density is increasing. Routine monitoring of exposure to alcohol outlets in communities could inform the planning, implementation, and evaluation of strategies to regulate alcohol outlet density, consistent with CPSTF recommendations (8).This approach to measuring alcohol outlet density, operationalized by assessing spatial access to alcohol outlets at the census block-level, can be replicated in other locations where address-level alcohol license data are available. Further studies, replicating such an approach, could help inform implementation of the CPSTF recommendation on regulating alcohol outlet density in other areas.

## References

[1] Centers for Disease Control and Prevention. Alcohol-Related Disease Impact (ARDI) application; 2013;http://apps.nccd.cdc.gov/DACH_ARDI/Default.aspx. Accessed October 16, 2014.

[2] Bouchery EE , Harwood HJ , Sacks JJ , Simon CJ , Brewer RD . Economic costs of excessive alcohol consumption in the US, 2006. Am J Prev Med 2011;41(5):516–24. 10.1016/j.amepre.2011.06.045 22011424

[3] Alcohol and crime: an analysis of national data on the prevalence of alcohol involvement in crime. Washington (DC): US Department of Justice; 1998.

[4] Livingston M . A longitudinal analysis of alcohol outlet density and domestic violence. Addiction 2011;106(5):919–25. 10.1111/j.1360-0443.2010.03333.x 21205052

[5] Livingston M , Chikritzhs T , Room R . Changing the density of alcohol outlets to reduce alcohol-related problems. Drug Alcohol Rev 2007;26(5):557–66. 10.1080/09595230701499191 17701520

[6] Mair C , Gruenewald PJ , Ponicki WR , Remer L . Varying impacts of alcohol outlet densities on violent assaults: explaining differences across neighborhoods. J Stud Alcohol Drugs 2013;74(1):50–8. 10.15288/jsad.2013.74.50 23200150 PMC3517264

[7] Treno AJ , Johnson FW , Remer LG , Gruenewald PJ . The impact of outlet densities on alcohol-related crashes: a spatial panel approach. Accid Anal Prev 2007;39(5):894–901. 10.1016/j.aap.2006.12.011 17275773

[8] Task Force on Community Preventive Services. Recommendations for reducing excessive alcohol consumption and alcohol-related harms by limiting alcohol outlet density. Am J Prev Med 2009;37(6):570–1. 10.1016/j.amepre.2009.09.021 19944926

[9] The Community Preventive Services Task Force. Preventing excessive alcohol consumption: privatization of retail alcohol sales [serial on the Internet]; 2011. http://www.thecommunityguide.org/alcohol/privatization.html. Accessed October 16, 2014.

[10] Gruenewald PJ , Remer L . Changes in outlet densities affect violence rates. Alcohol Clin Exp Res 2006;30(7):1184–93. 10.1111/j.1530-0277.2006.00141.x 16792566

[11] Xu Y , Yu Q , Scribner R , Theall K , Scribner S , Simonsen N . Multilevel spatiotemporal change-point models for evaluating the effect of an alcohol outlet control policy on changes in neighborhood assaultive violence rates. Spat Spatio-Temporal Epidemiol 2012;3(2):121–8. 10.1016/j.sste.2012.04.005 22682438 PMC6142809

[12] Yu Q , Scribner R , Carlin B , Theall K , Simonsen N , Ghosh-Dastidar B , Multilevel spatio-temporal dual changepoint models for relating alcohol outlet destruction and changes in neighbourhood rates of assaultive violence. Geospat Health 2008;2(2):161–72. 10.4081/gh.2008.240 18686265 PMC2995332

[13] Norström T . Outlet density and criminal violence in Norway, 1960–1995. J Stud Alcohol 2000;61(6):907–11. 10.15288/jsa.2000.61.907 11188497

[14] Grubesic TH , Pridemore WA . Alcohol outlets and clusters of violence. Int J Health Geogr 2011;10(1):30. 10.1186/1476-072X-10-30 21542932 PMC3098133

[15] Gorman DM , Speer PW , Gruenewald PJ , Labouvie EW . Spatial dynamics of alcohol availability, neighborhood structure, and violent crime. J Stud Alcohol 2001;62(5):628–36. 10.15288/jsa.2001.62.628 11702802

[16] Sampson RJ , Lauritsen JL . Violent victimization and offending: individual-, situational-, and community-level risk factors. In: Reiss J, Roth JA, editors. Understanding and preventing violence: social influences. Washington (DC): National Academy Press; 1994. p. 1–114.

[17] Bursik RJJ , Grasmick HG . Neighborhoods and crime: the dimensions of effective community control. New York (NY): Lexington Books; 1993.

[18] Hahn RA , Kuzara JL , Elder R , Brewer R , Chattopadhyay S , Fielding J , ; Task Force on Community Preventive Services. Effectiveness of policies restricting hours of alcohol sales in preventing excessive alcohol consumption and related harms. Am J Prev Med 2010;39(6):590–604. 10.1016/j.amepre.2010.09.016 21084080 PMC3712516

[19] Elder RW , Lawrence B , Janes G , Brewer RD , Toomey TL , Hingson RW , , editors. Enhanced enforcement of laws prohibiting sale of alcohol to minors: systematic review of effectiveness for reducing sales and underage drinking. Proceedings of Traffic safety and alcohol regulation: a symposium; 2007 June 5–6, Irvine (CA): Transportation Research Board of the National Academies; 2006.

[20] Henry S. Targeting Toungue and Groove: Buckhead nightclub learns it’s not healthy to stand in the path of progress. Creative Loafing. 2007 March 14.

[21] Sengupta S . Urbane Atlanta considers the color of its late-night revelry. New York Times. September 30, 2000. http://www.nytimes.com/2000/09/30/us/urbane-atlanta-considers-the-color-of-its-late-night-revelry.html. Acessed October 16, 2014.

[22] Atlanta City Council. Minimum age to enter nightclubs increased to 21. 2001; http://citycouncil.atlantaga.gov/2001/IMAGES/Adopted/1105/01O1671.pdf. Accessed October 16, 2014.

[23] Johnson J . Atlanta revelers may hear earlier last call. Los Angeles Times; 2003 November 23. http://articles.latimes.com/2003/nov/23/nation/na-buckhead23. Accessed October 16, 2014.

[24] Atlanta City Council. Last call moved back from 4:00 am to 2:30 am.; 2004. http://citycouncil.atlantaga.gov/2004/images/adopted/0216/04O0236.pdf. Accessed October 16, 2014.

[25] Franklin S , Pennington R . Fragile momentum: plan of action for rebuilding the Atlanta Police Department to help secure Atlanta’s position as capital of the new south; 2004. http://s3.amazonaws.com/zanran_storage/www.atlantapd.org/ContentPages/45790762.pdf. Accessed October 16, 2014.

[26] Atlanta City Council. Parking space requirement for nightclubs; 2004. http://citycouncil.atlantaga.gov/2004/images/adopted/1115/04O0044.pdf. Accessed October 16, 2014.

[27] Atlanta City Council. Anti-cruising ordinance; 2004. http://citycouncil.atlantaga.gov/2004/Images/Adopted/0105/Adopted.htm. Accessed October 16, 2014.

[28] Miller GA . The magical number seven plus or minus two: some limits on our capacity for processing information. Psychol Rev 1956;63(2):81–97. 10.1037/h0043158 13310704

[29] Britt HR , Carlin BP , Toomey TL , Wagenaar AC . Neighborhood level spatial analysis of the relationship between alcohol outlet density and criminal violence. Environ Ecol Stat 2005;12(4):411–26. 10.1007/s10651-005-1518-3

[30] Campbell CA , Hahn RA , Elder R , Brewer R , Chattopadhyay S , Fielding J , ; Task Force on Community Preventive Services. The effectiveness of limiting alcohol outlet density as a means of reducing excessive alcohol consumption and alcohol-related harms. Am J Prev Med 2009;37(6):556–69. 10.1016/j.amepre.2009.09.028 19944925

